# The Acti-Pair program helps men with prostate cancer increase physical activity with peer support: a mixed method pilot study

**DOI:** 10.3389/fpubh.2023.1321230

**Published:** 2024-01-08

**Authors:** Amandine Baudot, Nathalie Barth, Claire Colas, Maël Garros, Arnauld Garcin, Mathieu Oriol, Fanny Collange, Bienvenu Bongue, Frederic Roche, Franck Chauvin, Aurelie Bourmaud, David Hupin

**Affiliations:** ^1^National Institute of Health and Medical (INSERM) CIC1408 Centre d'Investigation Clinique Saint-Etienne, Saint-Etienne, France; ^2^Unité de recherche Clinique, Centre Hospitalier Universitaire de Saint-Etienne, Saint-Etienne, France; ^3^National Institute of Health and Medical Research (INSERM) U1059 SAnté INgéniérie BIOlogie, Saint-Etienne, France; ^4^Presage Institute - Université Jean Monnet, Saint-Etienne, France; ^5^Chaire santé des aînés - Université Jean Monnet, Saint-Etienne, France; ^6^Gérontopôle Auvergne Rhône-alpes, Saint-Etienne, France; ^7^Department of Clinical and Exercise Physiology, University Hospital Center of Saint-Etienne, Saint-Etienne, France; ^8^Sport-Health House, Departmental Olympic and Sports Committee of the Loire (42), Saint-Etienne, France; ^9^Centre Technique d'Appui et de Formation (CETAF), Saint-Etienne, France; ^10^National Institute of Health and Medical Research (INSERM) U1137 Infection, Antimicrobiens, Modélisation, Evolution, Paris, France; ^11^National Institute of Health and Medical Research (INSERM) CIC1426 Centre d'Investigation Clinique Robert Debré, Paris, France

**Keywords:** prostate cancer, exercise, motivation, maintenance, behavior mechanisms

## Abstract

**Background:**

Although the health benefits of physical activity (PA) are recognized, prostate cancer patients do not follow PA recommendations. Barriers to PA, whether physical, environmental or organizational, are known. Furthermore, even when these barriers are overcome, this achievement is not systematically accompanied by lifestyle change. Many strategies have shown to be effective in increasing patient adherence to PA. This study aims to assess the feasibility and the viability of the Acti-Pair program which combines three strategies: peer support, a personalized and realistic PA project, and support from health and adapted physical activity professionals in a local context.

**Methods and analysis:**

We conducted a pilot study utilizing a mixed qualitative and quantitative methodology, employing feasibility and viability assessments. Quantitative assessments included recruitment, retention adherence rates, process and potential effectiveness (PA and motivation) indicators; while qualitative methods were used to evaluate the program's practicality, suitability and usefulness. Indicators of potential effectiveness were assessed before and after the intervention using a Wilcoxon test for matched data. Qualitative data were collected through semistructured interviews conducted by two researchers with various program stakeholders. The study lasted for 3 years.

**Results:**

Twenty-four patients were recruited over a 25-month period. Forty-two percent of patients completed the program 3 months after the beginning. We recruited 14 peers and trained nine peers over a 10-month period. The program was coordinated extensively by adapted PA professionals, while health professionals were involved in recruiting patients and peers. Self-reporting of moderate to vigorous PA was increased after the Acti-Pair program initiation [42.86 (30.76) at baseline to 53.29 (50.73)]. Intrinsic motivation significantly increased after participation in the Acti-Pair program [1.76 (1.32) before the intervention vs. 2.91 (1.13) after the intervention]. The key player to support the Acti-Pair program in the field has been the PA support system. The main challenge has been the difficulty of health professionals in promoting PA.

**Discussion:**

This pilot study has shown that the Acti-Pair program is feasible and viable. It will allow us to extend the peer support intervention to other contexts and assess the effectiveness of this intervention and its generalization.

## Introduction

The beneficial effects of physical activity (PA) in the reduction of cancer mortality and the recurrence of cancer are widely recognized. Therefore, it is crucial for patients with prostate cancer to practice regular PA. For these patients with non-metastatic prostate cancer, 2.5 h of brisk walking per week (WHO recommendations) (1) is associated with a 29% decrease in cancer mortality (2) and a 57% reduction in recurrence (3). Moreover, PA can have significant effects on physical functioning, quality of life, maintenance of autonomy in tertiary prevention of prostate cancer (3, 4). However, despite the evidence, 60–70% of prostate survivors do not meet current public health guidelines of PA (5, 6).

In France, as well as in other European countries such as Germany, Italy, the United Kingdom, and Sweden, patients with chronic illnesses can receive adapted physical activity (APA) services through a medical prescription.^1^ These patients can then receive tailored support specific to their illness from a professional who is specifically trained in APA. In addition, many “Sport-Health” programs recently emerged in France for cancer patients have been developed in recent years.^2^ But in practice, adapted physical activity (APA) professionals observe few men in proposed sessions. There are numerous reasons why adherence to suggested programs is challenging amongst males with prostate cancer: (1) functional limitations (urinary incontinence, erectile dysfunction) (7, 8) due to side effects of prostate cancer treatments; (2) healthcare professionals do not regularly recommend PA to their patients for many reasons due to lack of awareness of PA, lack of specific training in prescribing PA during medical training, lack of priority for PA over chemotherapy or radiotherapy, and because it is not their role to talk to patients about PA (9, 10); and (3) PA programs are too often standardized (11).

Increasing prostate cancer patient' adherence to PA advice as part as of tertiary prevention is a challenge for personalized cancer care (12, 13). Informing people about the benefits does not allow them to change their lifestyle and thus integrate the PA into their daily life. The challenge is to find strategies that enable prostate cancer patients to practice regular PA and maintain it over time.

Social support has a positive impact on the quality of life for individuals with chronic diseases, especially cancer (14, 15). Additionally, it reduces cancer progression (16) and mortality risk (17). Social support has also been found to facilitate engagement in PA (18). Social support is defined as the assistance provided to individuals through their social connections with others, groups, and the broader community (19). It can assume various forms, such as emotional, informational and instrumental; and it can originate from formal or informal sources, including family, friends, community organizations, and professional support (20). Amongst forms of social support, peer support has already demonstrated its effectiveness in promoting regular PA levels in diabetic patients (21) (45), older individuals (22, 23), as well as more recently in breast cancer (24) and prostate cancer patients (25). With the help of peer intervention, patients can rely on social support to overcome psychological obstacles to PA identified in the literature, such as loss of self-esteem, lack of motivation and lack of competence. Another form of social support that has demonstrated its impact on PA is support from healthcare professionals (26, 27).

In order to encourage cancer patients to engage in and maintain regular physical activity, we need to take into account their preferences regarding the type of activity (18) in line with their representation of the practice, as well as tailoring the activity to the patient's needs and abilities. Program personalization has been shown to be effective in improving adherence to PA (26, 28).

To our knowledge, no study has combined several PA strategies: (1) personalized at home, (2) supported by healthcare professionals, and (3) supported by peers in the same program. A specific pathway for initiating and maintaining regular PA, involving all the players in the prostate cancer patient pathway should be considered.

A multidisciplinary team (composed of physicians, sociologists, a health promotion and prevention professional, and APA professionals) created the Acti-Pair program. This program combines three intervention strategies, each of which has independently demonstrated its effectiveness: (1) peer support (25, 29), and (2) personalized and realistic PA project (26, 30) (3) support from health and APA professionals (26, 27). The Acti-Pair program comprises multiple intervention strategies involving many stakeholders. When implementing the Acti-Pair program, it is crucial to assess whether it has effectively reached the target population, been integrated among various stakeholders, coordinated program activities, and perceived as useful by key stakeholders. The program's outcome may be influenced by the context, making it a complex intervention (31–33). The Medical Research Council (MRC) has suggested a methodological framework for assessing complex interventions (34). The initial step was to define the intervention's components, followed by testing its feasibility. To define intervention's components we have conducted a qualitative study, which has enabled us to identify the barriers and levers to regular PA by comparing two distinct populations in terms of PA: patients (inactive profiles) and peers (active profiles). This step has allowed us to refine the components of the intervention and its active ingredients. The results of the first step of the study have been published in separate article. Considering the feasibility of Acti-Pair program (second step suggested by the MRC) we conducted a pilot study at a local level in the Loire departement (France) both for prepare the evaluation and the intervention (35). A mixed qualitative and quantitative approach was used to assess the program's feasibility in terms of: 1/recruitment, retention and adherence criteria, as well as 2/its viability based on practical, suitable, helpful and evaluable criteria (36).

This article describes the results of this pilot study. The aim of this study was to assess the feasibility and viability of the Acti-Pair program for prostate survivors to improve PA in the local context of the Loire department in France.

## Materials and methods

### Study design and setting

This was a pilot study using a feasibility and viability evaluation using mixed quantitative and qualitative methodology. This methodology allows for the assessment of trial feasibility and evaluation of Acti-Pair's program viability in the context of implementation. Chen (36) has suggested assessing the viability of a multicomponent program in terms of whether it is practical, useful and supported by stakeholders (36). In other words, viability assessment involves evaluating if the program is adequately implemented, if its coordination is integrated and adapted to ongoing stakeholder activities, if it is evaluable, and if stakeholders have been successfully persuaded of its benefits with regard to PA practice. The intention is to gather input and experiences from stakeholders to demonstrate the program's practical and real-world setting (36) and to enable scaling of the program to a larger level (35, 37).

The study design has been previously described (38).

All participants have provided informed written consent to participate in the study according to the Declaration of Helsinki. The protocol was approved by the institutional review board (CPP Sud-Est I, France, 2018-A00710-55). The sponsor was Saint-Etienne University Hospital.

### Intervention: Acti-Pair program

The intervention was based on the theory of self-determination (39). This theory is based on the hypothesis that three psychological needs are at the basis of human motivation: the need for autonomy, the need for competence, and the need for social belonging (40). The Acti-Pair program is based on patients' preferences by establishing for each patient a personalized and realistic project outside of the medical system by supporting patients in disassociating from their illness (need for autonomy), added to motivational support carried out by peers (need for social belonging), followed by PA and health professionals who will enable the patient to acquire knowledge and skills about PA (need for competence). Motivation is part of a continuum of self-determination. This continuum presents several components of motivation ranging from: (1) motivation where the individual has no motivation and presents the lowest level of self-determination; (2) external regulation where the individual adopts a behavior by external pressure (threat or reward); (3) introjected regulation where the individual is motivated by feelings of shame and guilt about the behavior; (4) identified regulation where the individual identifies with the behavior and values it; and (5) integrated regulation where the behavior is performed in accordance with an individual's values. This last step corresponds to the highest level of self-determination and intrinsic motivation.

This theory has not been widely used but provides promising insights into the motivational mechanisms that explain PA engagement and behavior maintenance (18).

In order to implement the Acti-Pair program, we formed a partnership with the PA support system (support Device for Adapted Physical Activity Practice, DAPAP) of the Loire department.^3^ The objective of this support process has been to reinforce links between networks, health professionals, public and sports associations to facilitate the resumption and maintenance of PA for people who are not physically active and/or experiencing health difficulties.

#### Peers recruitment and training

We aimed to recruit five peers. Peers were recruited between August 2018 and June 2019 during follow-up visits for their prostate cancer with several specialists (urologists, oncologists, and radiotherapists) practicing in Lucien Neuwirth Cancer Institute or in Saint-Etienne University Hospital center. Peers were over 18 years old, with prostate cancer diagnosed at least 1 year ago, were not undergoing treatment (except hormone therapy), and were physically active according to WHO recommendations [more than 150 min of moderate-to-vigorous physical activity (MVPA) per week]. All peers provided written informed consent before being recruited in the study. Peers who agreed to participate in the study were asked to complete questionnaires: to evaluate their socioeconomic conditions (Evaluation of Precariousness and health Inequalities in Centers of health Examination, EPICE) (41), PA and sedentary behavior (Adult PA Questionnaire, APAQ, Jean Monnet University, Saint- Etienne, France) (38), and their motivation to practice PA (behavioral regulation in exercise questionnaire, BREQ-2) (42). They were also asked to wear an accelerometer on their non-dominant wrist (GT3X+, ActiGraph LLC, Pensacola, FL) for seven consecutive days to obtain an objective measure of their PA and sedentary behavior.

They were coached by a pluridisciplinary team composed of a sports physician, a sociologist, a health promotion professional and an APA professional. The description of the training provided was described in the study protocol (38). The objective of this training program was the acquisition of the following: (1) skills in PA counseling techniques (empathy, active listening) (provided by a sport psychologist); (2) the understanding of functional signs or symptoms that might indicate a medical problem in order to ensure patient safety (provided by a sports physician); and (3) the understanding required to determine heart rate and perceived exertion rate in order to ensure an appropriate level of effort (provided by an APA professional). We asked peers about their preferences concerning the modalities of accompaniment, as well as the criteria for pairing up. After the training, peers were also asked to complete a training feedback questionnaire. This questionnaire contained several items assessing satisfaction with the training using a 5-point Likert scale. The dimensions of the questionnaire assessed interest in the training, then the content in terms of duration, activities carried out, richness of exchanges, provision of information, quality of facilitation, user-friendliness, and acquisition of skills to become a peer. Each item was scored from 1 to 5 (1 for the most positive items and 5 for the most negative items). The scores were averaged to give an overall level of satisfaction with the training.

#### Patients recruitment and progress

Patients were recruited between May 2019 and June 2021 during follow-up visits for their prostate cancer with several specialists (urologists, oncologists, and radiotherapists) practicing in Lucien Neuwirth Cancer Institute or in Saint-Etienne University Hospital center. Patients were over 18 years old, with prostate cancer diagnosed at least 1 year ago, were not undergoing treatment (except hormone therapy), and were physically inactive according to WHO recommendations (<150 min of MVPA per week). All patients provided written informed consent before being recruited in the study.

**Figure 1:**
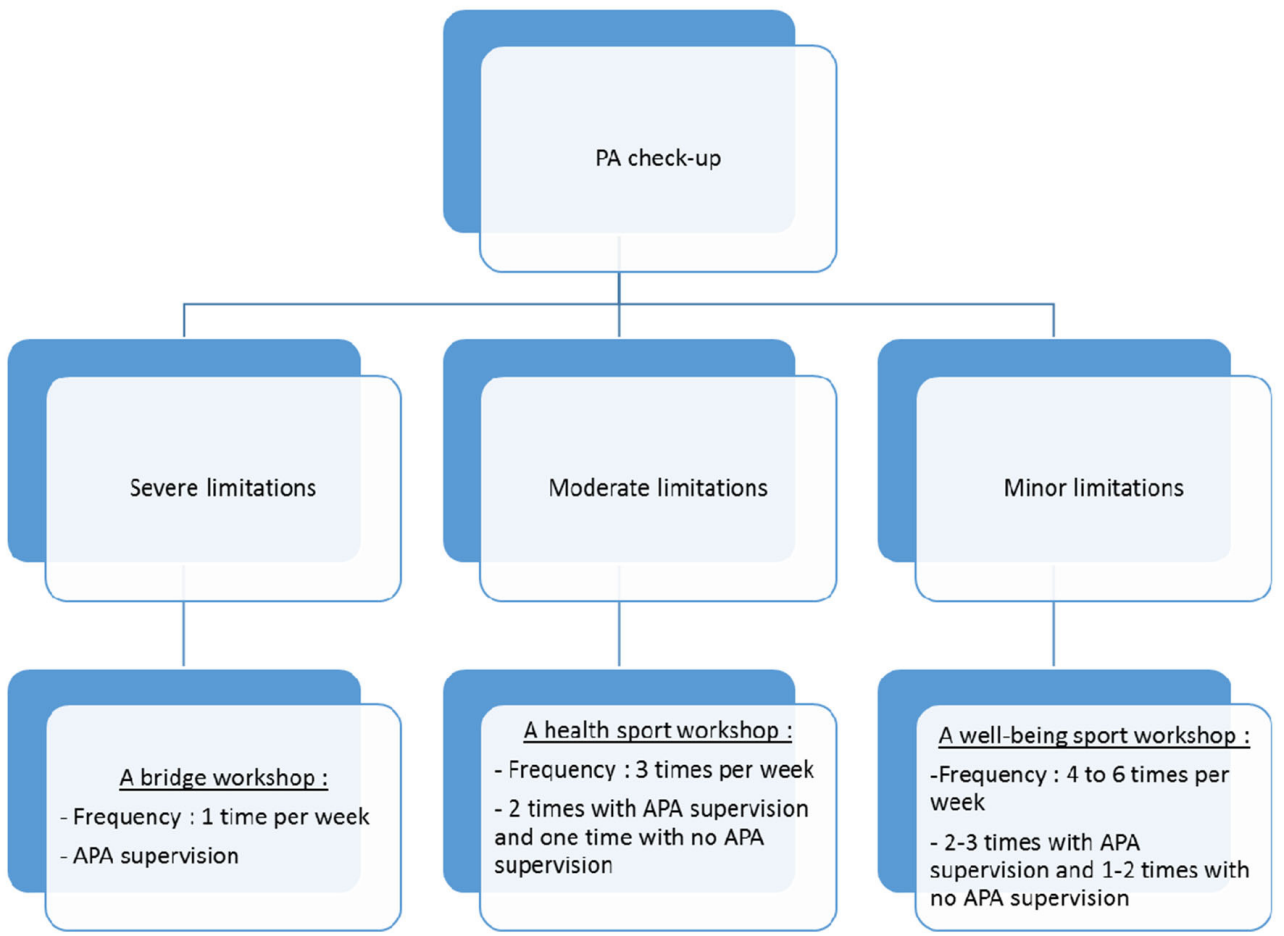
Patients programs proposals.

Patients who agreed to participate in the study were asked to complete a socioeconomic assessment questionnaire (EPICES) (43), PA and sedentary behavior (APAQ) (38), and their motivation to practice PA (BREQ-2) (42). They were also asked to wear an accelerometer for seven consecutive days (GT3X+, ActiGraph LLC, Pensacola, FL) to obtain an objective measure of their PA and sedentary behavior. Then a clinical research assistant (CRA) would forward patients' contact information to the DAPAP coordinator to complete the PA checkup. The DAPAP professional contacted patients to set up an appointment either at DAPAP or at the regional institute of sports medicine and engineering (IRMIS) localized at Saint-Etienne. During this PA checkup, the APA professional evaluated patients' physical capacities: (i) the 6-min walk test (6 MWT) used to assess aerobic capacity and endurance, (ii) the timed up and go test which assesses functional mobility, (iii) leg and arm strength tests, (iv) flexibility tests, and (v) postural balance. Afterwards, they talked together to assess the patient's desires and motivations in order to propose a 3-month PA program, which corresponded to their desires and abilities. It would be in the form of a workshop: (1) A bridge workshop (on medical prescription) for people with moderate to severe functional limitations: The beneficiaries were received over a period of 16 weeks, once a week for 1 h 15 (1 h of practice and 15 min of discussion) in groups of 5–10 persons. An evaluation was systematically done before the beginning of workshops and after 16 weeks. (2) A health or wellbeing sport workshop: The beneficiaries were received with moderate or minor limitations, in groups of up to 15 people (usually between 12 and 15), and for an unlimited period. Physical tests were not mandatory. This depended on the demands of the structures offering health sports and also on the will of the patients to undergo such tests. The choice of structure offering health sports was then made according to the range of APA workshops available, as close as possible to the patient's home. The patient would then go to the workshop to which he was referred in order to complete the PA program proposed by the DAPAP's APA professional (a bridge workshop or a health or wellbeing sport workshop). The criteria used to refer to the different programs and what they contain are shown in Figure 1.

The matching between peer and patient was done by a CRA. Then the CRA gave the peer's contact information to the patient and inversely, asking them to call each other and agree upon the terms of the accompaniment (frequency, physical presence or telephone support or other, and the location of the physical presence).

After completion of the PA programme, patients were asked to complete APAQ and BREQ-2 questionnaires and to wear another accelerometer for seven consecutive days. Patients and peers were also asked to complete a feedback questionnaire about the Acti-Pair program.

### Outcomes assessments

#### Feasibility

To assess the feasibility of the intervention, we assessed patients' recruitment, retention and adherence to the intervention by measuring the number of recruited patients, the number of patients dropping out of the program and the number of patients continuing the Acti-Pair program 3 months after its initiation.

We also assessed:

- Means indicators: number of peers, rate of trained peers, rate of mobilized structures, type of staff involved in peer training, number of trainings and time spent on training, number and type of involved professionals, training of APA and health professionals involved in the Acti-Pair program, number of assessments and duration
- Process indicators: terms and conditions for performing the assessments, modalities for PA professionals and peers support, terms and conditions of the training, peers and patients enrolment procedures, matching process, type of PA workshop, and peers and patient's feedback.

#### Potential effectiveness

To assess the potential effectiveness of the intervention, we used objective measurement of PA (in min/week), objective measurement of sedentary time (in h/d) via accelerometry (GT3X+, ActiGraph LLC, Pensacola, FL), and subjective complementary measurements of PA and sedentary periods using the APAQ questionnaire. We also assessed the motivation to practice PA with the BREQ-2 questionnaire.

#### Viability of the Acti-Pair program

The viability was analyzed with a qualitative method which analyzed conditions under which the intervention functioned. The analysis was focused on factors influencing implementation of the Acti-Pair program: Is the program adequately implemented? Does it fit into the routine organization of the various stakeholders? Does it benefit prostate cancer patients?

This assessment was conducted by semidirective interviews with health professionals and APA professionals, representative of sportshealth networks. A PhD sociologist and a PhD student trained in qualitative research conducted the interviews. All stakeholders, including nine health professionals and six APA professionals, were invited to participate in this qualitative phase.

### Data analysis plan

#### Quantitative analysis

All collected individual variables have been described by frequency (%) for categorical variables, and mean (SD) for quantitative variables.

The feasibility of the intervention was measured by frequency (%) of patients who continued the Acti-Pair program after 3 months.

Variations in MET-h/week for MVPA, h/week for sedentary, and motivation to practice PA have been assessed before and after patient intervention for patients and for peers before and at the end of the study. These comparisons have been made using a Wilcoxon test for matched data.

A comparison between peers' and patients' PA, sedentary behavior and motivation to practice PA was made. These comparisons were made using a Student *t*-test. Results have been considered to be significant at the 5% level (*p* > 0.05).

#### Qualitative analysis

Firstly, recorded interviews were transcribed verbatim. Two researchers (a PhD sociologist and a PhD student) had read transcripts in their entirety, and then line-by-line to extract relevant statements from the interviews following established guidelines for a thematic analysis (44). These statements were used to generate initial codes, and each transcript was then coded using this thematic coding scheme. The themes emerging from the first interviews have helped to refine the interview guide used for the next round of interviews. Data analysis was performed simultaneously with *N-Vivo software* (*QSR international, Burlington, USA*) and continuously with data collection to identify data saturation. The gathered information was categorized independently, by a PhD sociologist and a PhD student trained in qualitative research, into five main themes based on the objectives of the study. Coding and extracted themes were discussed and interpreted by the two researchers and a sports physician. These themes were subsequently specified and arranged into a logically consistent and coherent account. This account was supported by verbatim illustration.

The Clinical Research Unit (URC) from the Saint-Etienne University Hospital center (AG and AB) were in charge of monitoring and analyzing the data.

## Results

### Peers' and patients' characteristics

Peers were from 58 to 78 years old with a median age of 67 (±6.59) years old. Of the 14 peers recruited, only one patient was older than 75 years. Fifty-seven percent of patients lived in rural areas. The majority of peers lived with a partner and had a child. Peers characteristics are provided in Table 1. Only one peer was identified as precarious according to the EPICES score.

**Table 1:** Patient's and peer's characteristics.

	**Patients N (%)/M (±SD)**	**Peers N (%)/M (±SD)**
**Age (years)**	**69.96 (6.59)**	**68.05 (5.60)**
Age ≥ 75 years	5 (21%)	1 (7%)
BMI	27.62 (3.26)	24.71 (1.6)
**Place of living**		
Urban area	15 (68.18%)	6 (43%)
Rural area	7 (31.82%)	8(57%)
**Lives with**		
Alone	0 (0%)	1 (7%)
In couple with child(ren)	4 (14%)	1 (7%)
In couple without child(ren)	17 (77.27%)	12 (86%)
Other: friend, community living, …	1 (4.55%)	0 (0%)
Child(ren)	17 (89.47%)	13 (93%)
**Education**		
Stop before 14 years old	6 (37.5%)	1 (8%)
Stop before the high school diploma	4 (25%)	7 (58%)
Baccalaureate and more	1 (6.25%)	3 (25%)
After high school diploma	5 (31.25%)	1 (8%)
**Employment situation**		
Employed or self-employed	3 (15%)	1 (7%)
Retired	17 (85%)	13 (93%)
At home	0 (0%)	0 (0%)
Student	0 (0%)	0 (0%)
Job seeker	0 (0%)	0 (0%)
**Socio-occupational category**		
Farmer, operator	1 (5.56%)	0 (0%)
Craftsman, merchant, company manager	3 (16.67%)	1 (8%)
Executive, higher intellectual profession	5 (27.78%)	4 (31%)
Intermediate profession, teacher, foreman, technician, administrative	4 (22.22%)	4 (31%)
Clerk	3 (16.67%)	4 (31%)
Worker	2 (11.11%)	0 (0%)
**Precariousness**		
EPICE score	23.37 (18.99)	7.99 (10.18)
Precarious (EPICE score ≥ 30)	6 (27.27%)	1 (7%)

Patients were from 56 to 82 years old with a median age of 70 (±6.59) years old. Of the 24 patients recruited, five patients were older than 75 years. Sixty-eight percent of patients lived in urban areas and 32% of patients lived in rural areas. The majority of patients lived with a partner and had a child. Six patients were identified as precarious according to EPICES score, representing 27% of the patient sample. Patients' and peers' characteristics are provided in Table 1.

Patients and peers had a median duration of cancer diagnosis of 3 years. The majority of patients and peers has received surgery and/or radiation therapy as part of their cancer treatment. Five patients had received chemotherapy vs. only 1 peer. Patients' and peers' treatments are presented in Table 2.

**Table 2:** Patient's and peer's treatments.

	**Patients N (%)/M (±SD)**	**Peers N (%)/M (±SD)**
**Surgery**	**9 (37.5%)**	**8 (57%)**
Time from surgery to enrolment (in months)	53.6 (36.78)	83.68 (76.35)
**Radiotherapy**	13 (54.17%)	5 (36%)
Time from radiotherapy to enrolment (in months)	31.74 (25.69)	79.91 (100.02)
**Brachytherapy**	1 (4.17%)	2 (14%)
Time from brachytherapy to enrolment (in months)	152.07	48.02 (53.90)
**Chemotherapy**	5 (20.83%)	1 (7%)
Time from chemotherapy to enrolment (in months)	34.63 (38.22)	10.95
**Hormonotherapy**	19 (79.17%)	4 (29%)
Still in progress at the time of inclusion	13 (68%)	2 (14%)
Time from hormonotherapy to enrolment (in months)	22.09 (31.82)	16.03 (10.62)
**Active monitoring**	0 (0%)	2 (14%)
Time from active monitoring to enrolment (in months)		92 (67.14)

### Recruitment, retention, and adherence

**Figure 2:**
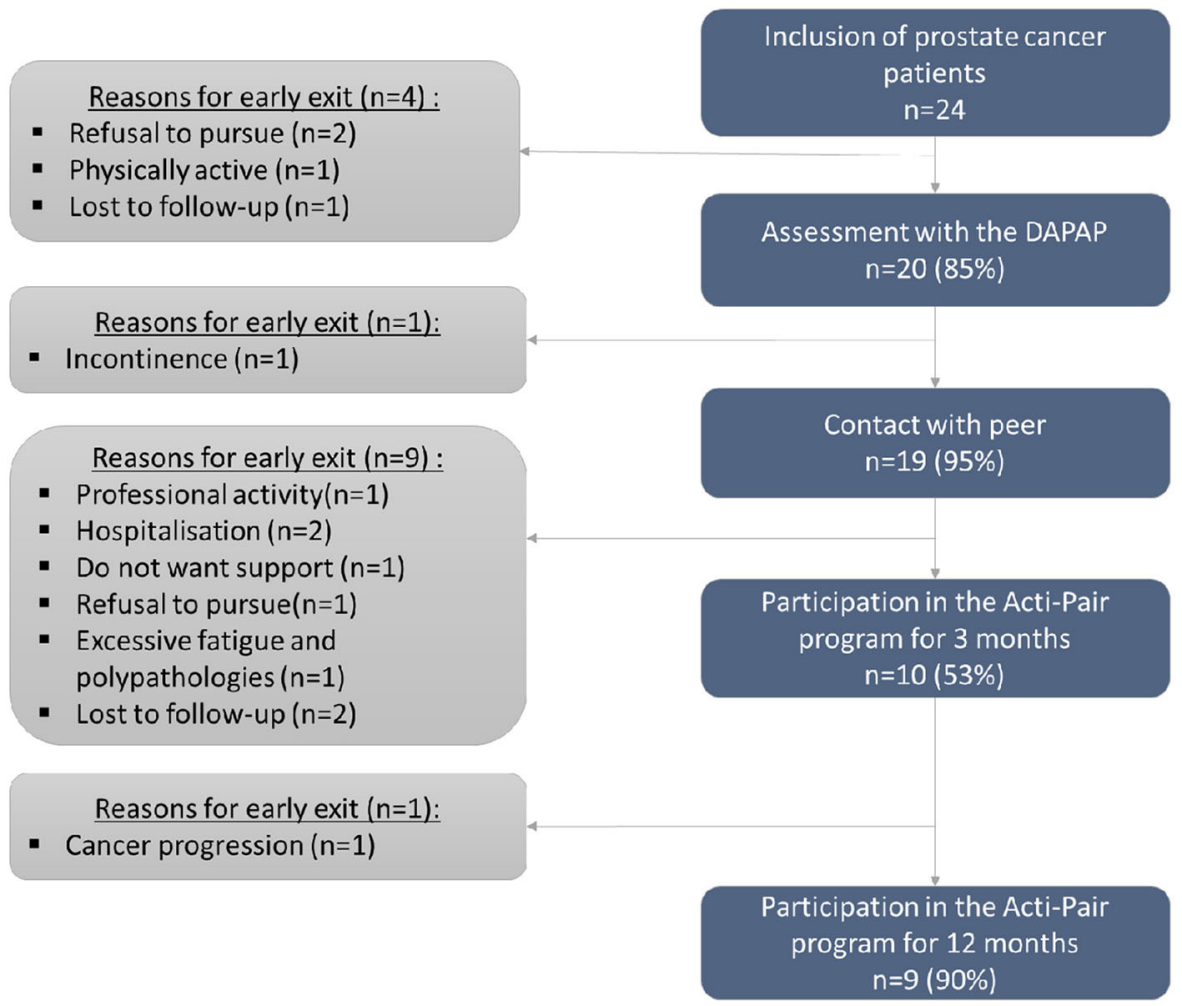
Patients' flow-chart.

Twenty-four patients were recruited over a 25-month period. Patients flow is provided in Figure 2.

Forty-two percent of patients had completed a PA project for at least 3 months. Main reasons for discharge were due to comorbidities associated with the pathology (fatigue, side effects of treatments). Reasons for withdrawal are presented in Figure 2.

### Means indicators

#### Peers

Fourteen peers were recruited over a 10-month period. Nine peers (64%) were trained to acquire the knowledge necessary to provide motivational support and coaching for patients' PA. Two training sessions were done: one session in May 2019 and another in September 2019. Each session lasted 3 h.

#### Professionals

Six APA professionals were implicated in the Acti-Pair program via the partnership with the DAPAP. Among APA professionals, one coordinator was in charge of coordinating the Acti-Pair program with the activities of the DAPAP. Two APA educators have completed twenty APA assessments. The duration of each assessment was ~1 h.

Nine health professionals were implicated in the Acti-Pair program to recruit patients and peers. Only specialists were implicated: two oncologists, three radiotherapists, two urologists, and one sports physician. No medical prescriptions were issued for the program. No general practitioner initiated the recruitment.

### Process indicators

#### Training

The training lasted 3 h with different workshops set up. (1) A presentation of the trainers with an icebreaker tool to allow participants to introduce themselves. (2) A presentation of the results of the first step of the program allowed the trained peers to identify the differences in profiles between themselves and patients they assist. (3) A brainstorming workshop to develop motivational follow-up skills. The following topics were discussed: How to motivate inactive patients to start and/or maintain regular PA? What are your fears and needs with regard to support? (4) Then they cobuilt the matching process and support modalities for the intervention. (5) They finished with a practical APA session with the APA and health professionals to get knowledge on functional signs (symptoms) that may indicate a medical problem to ensure patient safety and to learn how to examine heart rate and rate of perceived exertion to ensure level of effort. (6) Trained peers completed the formation by filling out a feedback questionnaire.

During the co-construction workshop, peers expressed the need to see patients accompanied at least during the first session. They also expressed the priority need for geographical proximity with the accompanied patient. The need to share the same treatment experience was expressed in a consensual manner amongst the trained peers as a secondary matching criterion.

A high level of satisfaction with the training was obtained among participants (mean score of 1.8 ± 0.68) and trainers (mean score of 1.57 ± 0.5).

#### DAPAP's role in the Acti-Pair program

The PA check-up was integrated into the DAPAP's usual practice to carry out: (1) an in-person interview to determine the patient's physical condition, motivations and wishes; (2) referral to adapted structures that offer an activity adapted to the patient's wishes and abilities, with the possibility of being accompanied by an APA professional during the first session; and (3) a regular follow-up over 2 years.

#### Modalities of matching process and support

The matching process was carried out by the research team. The geographical proximity between the peer and the accompanied patient was preferred. A secondary criterion was the similarity of the treatments received.

In terms of support arrangements, peers accompanied between 1 and 4 patients. The first meetings were all held in person. Some peers favored mixed (physical and phone) coaching, while others favored phone-only coaching, and still others wanted to provide both phone and physical coaching. Peer meetings were scheduled semi-annually beginning in May 2019 and November 2019 and were discontinued in March 2020, based on various COVID-19 related health measures.

#### Acti-Pair program's satisfaction

Patient's and peer's satisfaction were assessed by considering different aspects: the practical organization of the pairing, the type of PA practiced, and the benefits that the Acti-Pair participants received. Seven peers and seven patients answered the satisfaction questionnaire. In the majority of pairs, the peer contacted the patient. The frequency of interactions varied between pairs, ranging from several times a week to only once or twice a month. Some pairs did not have an interaction and in these cases, patients left the Acti-Pair program. The quality of the interactions was judged by the majority of respondents as very good to normal, with one peer rating the relationship with the patient as bad.

#### Type of PA practice

All peers reported regular PA, with a lot of outdoor activities, accompanied or alone. In 43% of the pairs interviewed, the peers took action to encourage their partner to join a club or gym. In the majority of cases (57%), the peer encouraged the accompanied patient to join him at their usual PA practice and in 75% of cases, the accompanied patient adhered to the peer's practice. Seventy-one percent of the peers reported a benefit. Seventy-one percent of peers reported that they did not maintain contact during the COVID-19 outbreak. Seventy-one percent of the peers declared that the Acti-Pair program had an impact on the human relationship, 29% on experience sharing, 29% a physical impact, and 29% a psychological impact. Peers appreciated the support of the project supervisors as well as the meetings with other peers.

A majority of practices took place in a sports club. The main reasons for PA practice was competition and leisure. With the Acti-Pair program, APA educators referred patients to bridge workshops, wellness workshops and health workshops.

### Potential effectiveness

#### Change in PA

At baseline, subjective measure of MVPA was significantly different between peers and patients (*p* = 0.0446), as well as sedentary time assessed with accelerometer (*p* = 0.0034) and APAQ questionnaire (*p* = 0.0120). Results are presented in Table 3.

**Table 3:** Comparison of peer and patients' PA at baseline.

**Physical activity (min/week)**	**Peers**	**Patients**	***p*-value**
**Accelerometer**	***N*** = **10**	***N*** = **17**	
Light intensity	196.72 (52.36)	196.02 (55.87)	0.9743
Moderate intensity	43.15 (22.44)	43.15 (22.44)	0.1512
Vigorous intensity	2.60 (3.38)	0.86 (0.90)	0.1432
MVPA	62.83 (30.73)	44.01 (23.06)	0.1140
Sedentary	550.04 (70.93)	627.56 (119.49)	**0.0446** ^*^
**APAQ**	***N*** = **9**	***N*** = **24**	
MPVA (min/week)	102.72 (44.41)	42.86 (30.76)	**0.0034** ^*^
Sedentary (h/week)	16.69 (14.51)	39.45 (34.32)	**0.0120** ^*^

^*^p <0.05.

The Acti-Pair program had increased self-report MVPA from 42.86 (30.76) at baseline to 53.29 (50.73) at the end of the intervention. We observed a decrease in objective measure of MVPA between enrolment and 12 months after the program [44.01 (23.06) at baseline vs. 22.23 (13.73) at the end of the intervention]. Results are presented in Table 4.

**Table 4:** Comparison of patients' PA before and after intervention.

**Patients' physical activity (min/week)**	**Before intervention**	**After intervention**	***p*-value**
**Accelerometer (min/week)**	***N*** = **17**	***N*** = **5**	
Light intensity	196.02 (55.87)	195.41 (82.66)	0.8927
Moderate intensity	43.15 (22.44)	22.12 (13.70)	0.5002
Vigorous intensity	0.86 (0.90)	0.11 (0.10)	0.5002
MVPA	44.01 (23.06)	22.23 (13.73)	0.5002
Sedentary	627.56 (119.49)	637.61 (82.68)	0.6858
**APAQ**	***N*** = **24**	***N*** = **8**	
MPVA (min/week)	42.86 (30.76)	53.29 (50.73)	0.1614
Sedentary (h/week)	39.45 (34.32)	37.5 (25.91)	0.3621

#### Change in motivation to practice physical activity

For patients, we noticed an increase in the relative autonomy index between the beginning and the end of the Acti-Pair program. If we distinguish the different elements of motivation, we observed an increase in introjected, identified and intrinsic motivation, with a significant difference concerning the evolution of intrinsic motivation during the Acti-Pair program (*p* = 0.035). Results are presented in Table 5.

**Table 5:** Comparison of patients' motivation to practice PA before and after intervention.

**Questionnaires**	**Before intervention**	**After intervention**	***p*-value**
**BREQ-2**	***N*** = **15**	***N*** = **8**	
Relative autonomy index (RAI)	5.67 (5.68)	10.53 (4.18)	0.2076
Amotivation	0.44 (0.59)	0.5 (0.8)	0.6547
External	0.64 (0.99)	0.81 (0.48)	0.7781
Introjected	0.99 (1.09)	1.38 (1.33)	0.4461
Identified	1.97 (1.13)	3.16 (0.63)	0.06735
Intrinsic	1.76 (1.32)	2.91 (1.13)	**0.02728** ^*^

^*^p <0.05.

#### Viability of the Acti-Pair program

Seven interviews were conducted during the implementation of the Acti-Pair program with the actors involved in the program; six APA professionals and one health professional.

##### Utility

All professionals saw a real interest in the participation of prostate cancer patients in the Acti-Pair program in terms of physical and psychological health benefits.

*APA professional n*°*1: “I see a real interest in PA for these people to try to maintain their state of health as much as possible, to limit the problems that the pathology can cause.”*,.

*APA professional n*°*4: “This is something that is already important during prostate cancer, in terms of the treatments, to limit the fatigue...”*.

*APA professional n*°*5: “On the mental level there could be real benefits with cancer patients.”*

*Health professional n*°*7: “In a global objective of improving the quality of life in particular, it can eventually be an improvement of the side effects of the treatment as well as of the survival parameters.”*

Other professionals also identified a real social benefit for the patients.

*APA professional n*°*4: “The social side also I think is very important, there is a pathology, it allows to free oneself, why not to talk about it too.”*

*Health professional n*°*7: “He is totally happy because it has totally stimulated him.”*.

Peer support appeared to be an element that enhanced the sharing of experiences:

*APA professional n*°*1: “The fact of having a peer with whom to talk, to exchange, to tell a little bit about what they went through [...] on the psychological aspect it's really important.”*

*APA professional n*°*4: “It's a very good thing because asking for advice from someone who has already had the disease, who can finally understand us about certain complications, certain difficulties, certain things, about the treatment, about all the side effects.”*

This peer support was also perceived as a major motivational element regarding the practice of PA:

*APA professional n*°*2: “The person who practises PA is going to pull the person who doesn't practise towards them. It's going to... have a motivational role”*.

*APA professional n*°*3: “This system of accompaniment [...] it's really positive, it boosts the patients.”*

*Health professional n*°*7: “The somewhat innovative idea of using patients who already have prostate cancer but who are sportsmen or women [...] to motivate, coach and brief patients [...] who are less physically active at the outset.”*

##### Practicability and suitability

Coordination between health and APA actors seemed at first sight not to have been achieved, despite the Acti-Pair program.

The health professional indicated that the most natural referral would be to a physiotherapist.

*Health professional n*°*7: “There are some patients for whom we will say why not physical therapy or rehabilitation [...] because there you go... We realise that we have physiotherapists who are somewhat specialised, that we now have good addresses. And it's true that we refer people to this or that person.”*

The health professional mentioned a medical prescription to optimize this referral but did not use it because of a general lack of knowledge about how it works.

*Health professional n*°*7: “I don't prescribe physical activity on a prescription [...] we, doctors, aren't very briefed on this either, i.e. I don't even know what to write, the consequences it has in terms of possibilities for the patient.”*

This professional, despite his participation in the Acti-Pair program, was not aware of the DAPAP and its functioning.

*Health professional n*°*7: “This is a contact that we can give to the patient or is it a prescription?”*

However, the health professional mentioned some barriers to the implementation of the Acti-Pair program. The first barrier identified was related to the health context of COVID-19, which clearly had an impact on patients' PA, especially those involved in the Acti-Pair program:

*Health professional n*°*7: “The current health problem, well that's clear that it's obvious but it has redesigned everything even if maybe with visio or with things like that there are things that are feasible”*

The second barrier mentioned was the need for geographical proximity between the patient and the peer:

*Health professional n*°*7: “It's certain that if the peer lives 2 km from the patient with whom he has to work, it makes things easier, whereas if he has one in [cities 75 km away], I don't know, it's much more difficult.”*

The third barrier mentioned is the motivation of patients to practice:

*Health professional n*°*7: “There are patients who are not at all sporty [...]. These are the patients I am not sure will be able to take it all in, although they are the ones who would really need it.”*

## Discussion

The implementation of the Acti-Pair program has demonstrated its feasibility in peers' recruitment and patients' adherence to the program. The program has shown a potential effectiveness on self-report of moderate to vigorous PA and intrinsic motivation. The PA support system is the key actor in the field to support the Acti-Pair program.

Initially, we hypothesized that we could recruit and train five peers within the 8-month recruitment period we set from a feasibility perspective. However, we ended up recruiting 14 peers and training 9 of them over a 10-month period. To achieve our goals, we required a larger peer workforce. So we recruited and trained more peers. The recruitment was carried out exclusively by specialist physicians. Of the 24 patients recruited, 42% continued the program after its initiation. This result is lower than in other studies involving peer motivational support for prostate cancer patients (25) or breast cancer patients (24). The majority of the reasons given were due to factors that could not be changed in the current program structure: lack of time, hospitalizations or polypathologies, or refusal to be accompanied by a peer. Moreover, the Acti-Pair program was conducted during the specific situation of the COVID-19: only three pairs were matched before the COVID-19 health crisis, which had a strong impact on support and on the practice of PA (45). The closure of clubs, associations has resulted in the cessation of supervised APA (46), hence preventing people from practicing their PA in a supervised way. In addition, the various health measures implemented prevented people from meeting and thus slowed down the setting up of new pairs. The anxiety-provoking nature of the various lockdowns also caused some social isolation (47) and thus created a rupture in the peer support dynamic and the link with the research team. Other elements of the program implementation have demonstrated its feasibility. Only one patient left the program after 3 months due to cancer progression. The challenge was therefore to reinforce the support during the first 3 months. Given the context of the implementation of the Acti-Pair program, we considered the program to be feasible.

This study allowed us to assess coordination between all stakeholders of the Acti-pair program in the management of patients with prostate cancer. The link with peers seemed to be well-accepted by APA professionals who saw it as an advantage, particularly in terms of motivational support. The health professional interviewed revealed a lack of knowledge of devices such as the DAPAP and would rather refer patients to a paramedical profession such as physiotherapist. Initially, we wanted to include general practitioners in the framework of PA's prescription. The recruitment was carried out exclusively by specialist physicians. The link between general practitioners, specialist physicians and DAPAP seemed difficult to obtain in order to integrate the Acti-pair program into the patient's cancer journey. In this pilot study, no PA's prescription was made by physicians to address their patients to DAPAP. However, the prescription facilitated the possibility of supervised PA and provided opportunities to practice PA or APA (47, 48). The lack of knowledge of existing programs was a barrier to the promotion of PA by health professionals that was well-known in the literature (49). Despite this, the link between health professionals and DAPAP was made exclusively in the framework of the pilot study. The DAPAP proved to be the key actor in the field to support the Acti-Pair program. DAPAP's APA professionals performed all the health assessments to guide patients to sports and health facilities close to their home, but also to allow them to choose an APA that is convenient for them. These assessments, which were part of DAPAP's usual practice, without representing an additional workload, contributed to the program's acceptability by APA professionals. The Acti-Pair program, through its coordination between different professionals, therefore appeared to be a lever for creating a link between the various professionals who care for prostate cancer patients and PA professionals who are able to help patients initiate PA and, above all, to maintain it on a regular basis.

Matching criteria were chosen by peer consensus during the training. They prioritized the geographic proximity between themselves and the patient they were supporting. As Jeffries pointed out, geographic proximity broke down the spatial barriers that inhibited peer support (50). Modalities of support were also diverse according to the pairs and demonstrated the need for flexibility, particularly concerning the frequency and nature of contacts (51). As a result, the peer-patient relationship is a crucial element of the support process and a fundamental aspect of the success of motivation peer support that this relationship is one of the bases for the success of motivational peer support (52). The therapeutic alliance requires emotional connection, empathy and open dialogue that can be provided through minimal training. Peer support is based on interpersonal relationships between people, involving the sharing of illness-related experiences (53). Satisfaction with the program showed the importance of social connection for both patients and peers in peer support, as raised many times in the literature (54).

The Acti-Pair program has shown increased self-report MVPA. These results were better than those obtained by Galvão et al. (25) in which PA of patients who received motivational support from a peer increased in the first 3 months, then decreased at 6 and 12 months (25). Results on self-report MVPA have not been found on objective MVPA. Higher self-reported PA compared to accelerometer measure has been noticed in older men (55). The questionnaire may indeed lead to a social desirability bias causing the person to overestimate his or her PA, hence the importance of coupling the subjective measurement with an objective measurement.

This study has shown the Acti-Pair program has increased the autonomous and intrinsic motivation to practice PA in prostate cancer patients. A systematic review of Teixeira et al. (56) has shown that the intrinsic motivation being more predictive of long-term exercise adherence (56). So the Acti-Pair program seems to be a good predictor of maintaining regular PA. A health professional has mentioned the importance of the step of behavioral change in which the patient is in and the likelihood of increasing social inequalities. It is highly likely that patients located in precontemplation or contemplation steps—i.e., who are either unaware of the problem or aware of the need to do PA but have not yet taken the step (57)—will not wish to participate in the Acti-Pair program. The Acti-Pair program in its current form does not address this issue, and a component for patients who are in the first two stages of the wheel of change, whom may be the patients with the greatest need to practice PA, needs to be included to prevent inequalities arising from the intervention (54). In order to reach patients in the contemplation and pre-contemplation stages, it would be interesting to develop the Acti-Pair programme by adding some strategies:

- A strategy combining both information and education aimed at patients to inform them about the benefits of PA for their health and their cancer (58).
- A strategy involving health professionals in promoting PA through short messages (59).

### Limitations

Our pilot study had several limitations. Firstly, the small sample size and the absence of a control group does not allow us to conclude on effects of the Acti-Pair program. Secondly, the restriction to a single department and therefore a specific organization does not enable the results to be extended to other contexts. Thirdly, health professionals did not respond favorably to requests for interviews. It is therefore necessary to make physicians aware of the practice of PA before the program begins, but also of the prescription, which appears to be an important element in the coordination of health and PA actors. In order to conclude on the effectiveness of the Acti-Pair program on the maintenance of regular PA for patients with prostate cancer, it will be necessary to set up a cluster stepped wedge randomized controlled trial that can account for the inherent variability of the field.

### Strengths

Although the study has limitations, its methodology is an important strength. With a mixed qualitative and quantitative design, we were able to evaluate the recruiting peers' feasibility, retention and adherence rates, and understand the perception those involved in the program. The viability evaluation allowed us to evaluate individual program components. Firstly, the practicability was assessed as existing organizations facilitated coordinating the Acti-Pair program and implementing intervention-related activities on a routine basis. Secondly, the suitability of the program was evaluated as APA and DAPAP professionals integrated it into their routine organization and utilized their expertise. Finally, professionals perceived the program as useful in terms of its benefit for patients.

This preliminary assessment of the Acti-Pair program's full scale will provide the means to evaluate its real-life effectiveness.

## Conclusion

Despite the limitations of this pilot study, our mixed-methods results introduce the Acti-Pair program as an innovative intervention to increase prostate cancer patient's PA. This pilot study has demonstrated the feasibility and viability of the Acti-Pair program in a specific setting for prostate cancer patients. This pilot study will then allow us to extend the Acti-Pair program to other contexts and evaluate the effectiveness of this intervention and its generalizability. In a French context, the results of this pilot study showed that a program composed of three strategies could be implemented for prostate cancer patients.

Further research is necessary to determine efficient methods for involving healthcare professionals in promoting PA since this technique was successful in encouraging patients to engage in regular PA.

## Data availability statement

The raw data supporting the conclusions of this article will be made available by the authors, without undue reservation.

## Ethics statement

The studies involving humans were approved by Comité de Protection des Personnes Sud-Est I, France. The studies were conducted in accordance with the local legislation and institutional requirements. The participants provided their written informed consent to participate in this study.

## Author contributions

ABa: Conceptualization, Formal analysis, Funding acquisition, Investigation, Methodology, Writing – original draft. NB: Conceptualization, Funding acquisition, Validation, Writing – review & editing. CC: Formal analysis, Investigation, Writing – review & editing. MG: Investigation, Writing – review & editing. AG: Project administration, Writing – review & editing. MO: Conceptualization, Writing – review & editing. FCo: Writing – review & editing. BB: Writing – review & editing. FR: Writing – review & editing. FCh: Writing – review & editing. ABo: Writing – review & editing. DH: Conceptualization, Funding acquisition, Methodology, Supervision, Writing – review & editing.

## Footnotes

^1^JORF n°0304 du 31 décembre 2016. Available online at: https://www.legifrance.gouv.fr/jorf/id/JORFTEXT000033748987.

^2^VIDAL. *Médicosport-santé*. Available online at: https://www.vidal.fr/sante/sport/infos-sport-medicosport-sante/.

^3^Portail du Sport Santé Bien-Être Auvergne-Rhône-Alpes - Les dispositifis d'accompagnement. Available online at: https://www.sport-sante-auvergne-rhone-alpes.fr/intermediaires/les-dispositif-daccompagnement/

## References

[1] Organisation mondiale de la Santé. Lignes directrices de l'OMS sur l'activité physique et la sédentarité. Organisation mondiale de la Santé (2022). Available online at: https://www.who.int/fr/news-room/fact-sheets/detail/physical-activity (accessed November 21, 2023).

[2] Bonn SE Sjölander A Lagerros YT Wiklund F Stattin P Holmberg E. Physical activity and survival among men diagnosed with prostate cancer. Cancer Epidemiol. Biomarkers Prevent. (2015) 24:57–64. 10.1158/1055-9965.EPI-14-0707 25527697

[3] Richman EL Kenfield SA Stampfer MJ Paciorek A Carroll PR Chan JM. Physical activity after diagnosis and risk of prostate cancer progression: data from the cancer of the prostate strategic urologic research endeavor. Cancer Res. (2011) 71:3889–95. 10.1158/0008-5472.CAN-10-3932 21610110 PMC3107352

[4] Schneider CM Hsieh CC Sprod LK Carter SD Hayward R. Cancer treatment-induced alterations in muscular fitness and quality of life: the role of exercise training. Ann Oncol. (2007) 18:1957–62. 10.1093/annonc/mdm364 17804476

[5] Blanchard CM Stein Kevin D Baker F Dent MF Denniston MM Courneya KS. Association between current lifestyle behaviors and health-related quality of life in breast, colorectal, and prostate cancer survivors. Psychol Health. (2004) 19:1–13. 10.1080/08870440310001606507

[6] Coups E Ostroff J. A population-based estimate of the prevalence of behavioral risk factors among adult cancer survivors and noncancer controls. Prev Med. (2005) 40:702–11. 10.1016/j.ypmed.2004.09.011 15850868

[7] Kyrdalen AE Dahl AA Hernes E Småstuen MC Fosså SD. A national study of adverse effects and global quality of life among candidates for curative treatment for prostate cancer. BJU Int. (2013) 111:221–32. 10.1111/j.1464-410X.2012.11198.x 22672151

[8] Gaskin CJ Craike M Mohebbi M Salmon J Courneya KS Broadbent S. Associations of objectively measured moderate-to-vigorous physical activity and sedentary behavior with quality of life and psychological well-being in prostate cancer survivors. Cancer Causes Control. (2016) 27:1093–103. 10.1007/s10552-016-0787-5 27469939 PMC4983284

[9] Spellman C Craike M Livingston P. Knowledge, attitudes and practices of clinicians in promoting physical activity to prostate cancer survivors. Health Educ J. (2014) 73:566–75. 10.1177/0017896913508395

[10] Nadler M Bainbridge D Tomasone J Cheifetz O Juergens RA Sussman J. Oncology care provider perspectives on exercise promotion in people with cancer: an examination of knowledge, practices, barriers, and facilitators. Support Care Cancer. (2017) 25:2297–304. 10.1007/s00520-017-3640-9 28258503

[11] Fox L Wiseman T Cahill D Beyer K Peat N Rammant E. Barriers and facilitators to physical activity in men with prostate cancer: a qualitative and quantitative systematic review. Psychooncology. (2019) 28:2270–85. 10.1002/pon.5240 31617635

[12] Blaney JM Lowe-Strong A Rankin-Watt J Campbell A Gracey JH. Cancer survivors' exercise barriers, facilitators and preferences in the context of fatigue, quality of life and physical activity participation: a questionnaire-survey. Psychooncology. (2013) 22:186–94. 10.1002/pon.2072 23296635

[13] Halbert CH. Social and clinical determinants of physical activity in prostate cancer survivors. Support Care Cancer. (2021) 29:459–65. 10.1007/s00520-020-05482-1 32394247 PMC7655513

[14] Chambers A Damone E Chen YT Nyrop K Deal A Muss H. Social support and outcomes in older adults with lung cancer. J Geriatr Oncol. (2022) 13:214–9. 10.1016/j.jgo.2021.09.009 34629320 PMC8970686

[15] Chollou KM Shirzadi S Pourrazavi S Babazadeh T Ranjbaran S. The role of perceived social support on quality of life in people with cardiovascular diseases. Ethiop J Health Sci. (2022) 32:1019–26. 10.4314/ejhs.v32i5.17 36262697 PMC9554781

[16] Nausheen B Gidron Y Peveler R Moss-Morris R. Social support and cancer progression: a systematic review. J Psychosomat Res. (2009) 67:403–15. 10.1016/j.jpsychores.2008.12.012 19837203

[17] Ikeda A Kawachi I Iso H Iwasaki M Inoue M Tsugane S. Social support and cancer incidence and mortality: the JPHC study cohort II. Cancer Causes Control. (2013) 24:847–60. 10.1007/s10552-013-0147-7 23549959

[18] INCA. Bénéfices De L' Activité Physique Pendant Et Après Cancer Des Connaissances Scientifiques Aux Repères Pratiques. France: Collection Etats des lieux et des connaissances (2017).

[19] Lin N Simeone RS Ensel WM Kuo W. Social support, stressful life events, and illness: a model and an empirical test. J Health Soc Behav. (1979) 20:108–19. 10.2307/2136433 479524

[20] Li F Luo S Mu W Li Y Ye L Zheng X. Effects of sources of social support and resilience on the mental health of different age groups during the COVID-19 pandemic. BMC Psychiatry. (2021) 21:16. 10.1186/s12888-020-03012-1 33413238 PMC7789076

[21] Tudor-Locke C Lauzon N Myers AM Bell RC Chan CB McCargar L. Effectiveness of the First step Programme delivered by professionals versus peers. J Phys Act Health. (2009) 6:456–62. 10.1123/jpah.6.4.456 19842459

[22] Buman MP. Peer volunteers improve long-term maintenance of physical activity with older adults: a randomized controlled trial. J Phys Act Health. (2011) 8:S257–66. 10.1123/jpah.8.s2.s257 21918240 PMC3181088

[23] Castro CM Pruitt LA Buman MP King AC. Physical activity programme delivery by professionals versus volunteers: the TEAM randomized trial. Health Psychol. (2011) 30:285–94. 10.1037/a0021980 21553972 PMC3092123

[24] Pinto BM Stein K Dunsiger S. Peers promoting physical activity among breast cancer survivors: a randomized controlled trial. Health Psychol. (2015) 34:463–72. 10.1037/hea0000120 25110844 PMC4441331

[25] Galvão DA Newton RU Girgis A Lepore SJ Stiller A Mihalopoulos C. Randomized controlled trial of a peer led multimodal intervention for men with prostate cancer to increase exercise participation. Psychooncology. (2018) 27:199–207. 10.1002/pon.4495 28685892

[26] Orrow G Kinmonth AL Sanderson S Sutton S. Effectiveness of physical activity promotion based in primary care : systematic review and meta-analysis of randomised controlled trials. Br J Sports Med. (2012) 1389:1–17. 10.1136/bmj.e1389 23243114

[27] Livingston PM Craike MJ Salmon J Courneya KS Gaskin CJ Fraser SF. Effects of a clinician referral and exercise programme for men who have completed active treatment for prostate cancer: A multicenter cluster randomized controlled trial (ENGAGE). Cancer. (2015) 121:2646–54. 10.1002/cncr.29385 25877784 PMC4654333

[28] Pinto BM Frierson GM Rabin C Trunzo JJ Marcus BH. Home-based physical activity intervention for breast cancer patients. J Clin Oncol. (2005) 23:3577–87. 10.1200/JCO.2005.03.080 15908668

[29] DeMello MM Pinto BM Mitchell S Dunsiger SI Stein K. Peer support for physical activity adoption among breast cancer survivors: do the helped resemble the helpers?. Eur J Cancer Care. (2018) 27:1–8. 10.1111/ecc.12849 29637645 PMC6084778

[30] Pinto BM Rabin C Abdow S Papandonatos GD. A pilot study on disseminating physical activity promotion among cancer survivors: a brief report. Psychooncology. (2008) 17:517–21. 10.1002/pon.1268 17847122

[31] Shiell A Hawe P Gold L. Complex interventions or complex systems? Implications for health economic evaluation. BMJ. (2008) 336:1281–3. 10.1136/bmj.39569.510521.AD 18535071 PMC2413333

[32] Hawe P Shiell A Riley T. Theorising interventions as events in systems. Am J Community Psychol. (2009) 43:267–76. 10.1007/s10464-009-9229-9 19390961

[33] Clark AM. What are the components of complex interventions in healthcare? Theorizing approaches to parts, powers and the whole intervention. Social Sci Med. (2013) 93:185–93. 10.1016/j.socscimed.2012.03.035 22580076

[34] Campbell M. Framework for design and evaluation of complex interventions to improve health. BMJ. (2000) 321:694–6. 10.1136/bmj.321.7262.694 10987780 PMC1118564

[35] Thabane L Cambon L Potvin L Pommier J Kivits J Minary L. Population health intervention research: what is the place for pilot studies? Trials. (2019) 20:1–6. 10.1186/s13063-019-3422-4 31146768 PMC6543677

[36] Chen HT. The bottom-up approach to integrative validity: a new perspective for programme evaluation. Eval Progr Plann. (2010) 33:205–14. 10.1016/j.evalprogplan.2009.10.002 19931908

[37] Cambon L Alla F. Current challenges in population health intervention research. J Epidemiol Commun Health. (2019) 73:990–2. 10.1136/jech-2019-212225 31315897

[38] Baudot A Barth N Colas C Garros M Garcin A Oriol M. The physical activity experience of prostate cancer patients: a multicentre peer motivation monitoring feasibility study. The Acti-Pair study Pilot and Feasibility Studies. BioMed Central. (2022) 8:12. 10.1186/s40814-022-00966-9 35063040 PMC8781045

[39] Ryan RM Deci EL. Self-determination theory and the facilitation of intrinsic motivation, social development, and well-being. Am Psychol. (2000) 55:68–78. 10.1037/0003-066X.55.1.68 11392867

[40] Milne HM Wallman KE Guilfoyle A Gordon S Corneya KS. Self-determination theory and physical activity among breast cancer survivors. J Sport Exerc Psychol. (2016) 30:23–38. 10.1123/jsep.30.1.23 18369241

[41] Sass Catherine et al. Le score Epices : un score individuel de précarité. Bull Epidémiol Hebd. (2006) 14:93–9.

[42] Markland D Tobin V. A modification to the behavioural regulation in exercise questionnaire to include an assessment of amotivation. J Sport Exerc Psychol. (2004) 26:191–6. 10.1123/jsep.26.2.191

[43] Sass C Guéguen R Moulin JJ Abric L Dauphinot V Dupré C. Comparison of the individual deprivation index of the French Health Examination Centres and the administrative definition of deprivation. Sante Publ. (2006) 18:513–22. 10.3917/spub.064.0513 17294755

[44] Braun V Clarke V. Using thematic analysis in psychology. Qual Res Psychol. (2006) 3:77–101. 10.1191/1478088706qp063oa

[45] Ammar A Brach M Trabelsi K Chtourou H Boukhris O Masmoudi L. Effects of COVID-19 home confinement on eating behaviour and physical activity: results of the ECLB-COVID19 International Online Survey. Nutrients. (2020) 12:1583. 10.3390/nu12061583 32481594 PMC7352706

[46] Colas C Jumel A Vericel M-P Barth N Manzanares J Goutte J. Understanding experiences of fibromyalgia patients involved in the fimouv study during COVID-19 lockdown. Front Psycho. (2021) 12:645092. 10.3389/fpsyg.2021.645092 34354626 PMC8329548

[47] Palmer K Monaco A Kivipelto M Onder G Maggi S Michel J-P. The potential long-term impact of the COVID-19 outbreak on patients with non-communicable diseases in Europe: consequences for healthy ageing. Aging Clin Exp Res. (2020) 32:1189–94. 10.1007/s40520-020-01601-4 32458356 PMC7248450

[48] Stahl T. The importance of policy orientation and environment on physical activity participation–a comparative analysis between Eastern Germany, Western Germany and Finland. Health Promot Int. (2002) 17:235–46. 10.1093/heapro/17.3.235 12147638

[49] Albert FA Crowe MJ Malau-Aduli AEO Malau-Aduli BS. Physical activity promotion: a systematic review of the perceptions of healthcare professionals. Int J Environ Res Public Health. (2020) 17:4358. 10.3390/ijerph17124358 32570715 PMC7345303

[50] Jeffries M Mathieson A Kennedy A Kirk S Morris R Blickem C. Participation in voluntary and community organisations in the United Kingdom and the influences on the self-management of long-term conditions. Health Soc Care Commun. (2015) 23:252–61. 10.1111/hsc.12138 25175423

[51] Ashrafi S Deo N Yip AKW Seddigh S Moradi R Waraich R. Autopsy of a telephone-based peer support intervention: exploring participants' perspectives of and experiences with a self-management support model for adults with type 2 diabetes from speciality care settings. Diabet Med. (2022) 39:e14924. 10.1111/dme.14924 36097326

[52] Adam O Horvat LSG. The Working Alliance: Theory, Research, and Practice, John Wiley & Sons (1994).

[53] Pistrang N Jay Z Gessler S Barker C. Telephone peer support for women with gynaecological cancer: benefits and challenges for supporters. Psychooncology. (2013) 22:886–94. 10.1002/pon.3080 22585444

[54] Lehne G Bolte G. Impact of universal interventions on social inequalities in physical activity among older adults: An equity-focused systematic review. Int J Behav Nutr Phys Act. (2017) 14:20. 10.1186/s12966-017-0472-4 28187766 PMC5303302

[55] Dyrstad SM Hansen BH Holme IM Anderssen SA. Comparison of self-reported versus accelerometer-measured physical activity. Med Sci Sports Exerc. (2014) 46:99–106. 10.1249/MSS.0b013e3182a0595f 23793232

[56] Teixeira PJ Carraça EV Markland D Silva MN Ryan RM. Exercise, physical activity, and self-determination theory: a systematic review. Int J Behav Nutr Phys Activity. (2012) 9:78. 10.1186/1479-5868-9-78 PMC3441783 22726453

[57] Nigg CR Burbank PM Padula C Dufresne R Rossi JS Velicer WF. Stages of change across ten health risk behaviors for older adults. Gerontologist. (1999) 39:473–82. 10.1093/geront/39.4.473 10495586

[58] Stonerock GL Blumenthal JA. Role of counseling to promote adherence in healthy lifestyle medicine: strategies to improve exercise adherence and enhance physical activity. Prog Cardiovasc Dis. (2017) 59:455–62. 10.1016/j.pcad.2016.09.003 27640186 PMC5350064

[59] Lamming L Pears S Mason D Morton K Bijker M Sutton S. What do we know about brief interventions for physical activity that could be delivered in primary care consultations? A systematic review of reviews. Prev Med. (2017) 99:152–63. 10.1016/j.ypmed.2017.02.017 28232098

